# Case report: Adult NTRK-rearranged spindle cell neoplasms with TPM3-NTRK1 fusion in the pelvic

**DOI:** 10.3389/fonc.2024.1308916

**Published:** 2024-01-31

**Authors:** Qiurui Cao, Zhifang Huang, Hong Liang, Xing Hu, Lucas Wang, Yaxian Yang, Bin Lian, Jian Huang, Jinyan Guo

**Affiliations:** ^1^ Department of Proctology, Wuyi Traditional Chinese Medicine Hospital, Jiangmen, Guangdong, China; ^2^ Precision Medicine Center, Guangzhou Huayin Health Medical Group Co., Ltd, Guangzhou, Guangdong, China

**Keywords:** NTRK-rearranged spindle cell neoplasms, molecular characteristic, TPM3-NTRK1, pathology, case report

## Abstract

NTRK-rearranged spindle cell neoplasms (NTRK-RSCNs) are rare soft tissue tumor molecularly characterized by NTRK gene rearrangement, which occurs mostly in children and young adults, and rarely in adults. The abnormal tumor located in superficial or deep soft tissues of human extremities and trunk mostly, and rarely also involves abdominal organs. In this case, we report a malignant NTRK-RSCN that occurred in the pelvic region of an adult. The patient was found to have a large tumor in the pelvic region with a pathological diagnosis of infiltrative growth of short spindle-shaped tumor cells with marked heterogeneity. Immunohistochemistry of this patient showed positive vimentin, pan-TRK and Ki67 (approximately 60%) indicators with negative S100, Desmin and DOG1. Molecular diagnosis revealed c-KIT and PDGFRα wild type with TPM3-NTRK1 fusion, unfortunately this patient had a rapidly progressive disease and passed away. This case highlights the gene mutation in the molecular characteristics of NTRK-RSCNs, and the significance of accurate molecular typing for the diagnosis of difficult cases.

## Introduction

Neurotrophic tyrosine receptor kinase (NTRK) fusions are pathogenetic alterations associated with various cancer types, including soft tissue sarcoma (STS), colorectal cancer, papillary thyroid cancer, glioma, secretory carcinoma, Spitz tumor and so on (1, 2). NTRK-rearranged spindle cell neoplasms(NTRK-RSCNs) are defined as an emerging entity of tumors according to the 5th edition of the World Health Organization (WHO) Classification of Soft Tissue and Bone Sarcomas (3). Most NTRK-RSCNs entities are of low-grade characterized, while little display a higher-grade phenotype, with markedly increased cellularity arranged in intersecting fascicles and a high mitotic count. Histologically, NTRK-RSCNs show a wide morphologic spectrum, usually with co-expression of CD34 and S100 and diffuse pan-TRK immunoreactivity (1, 4).

In this article, we report our diagnosis experience of an adult NTRK-RSCNs with TPM3-NTRK1 fusion in pelvic by the clinical, imaging, histopathological, and molecular characteristics, illustrating the role of integrated pathological diagnosis in rare and difficult tumors.

## Case report

**Figure 1:**
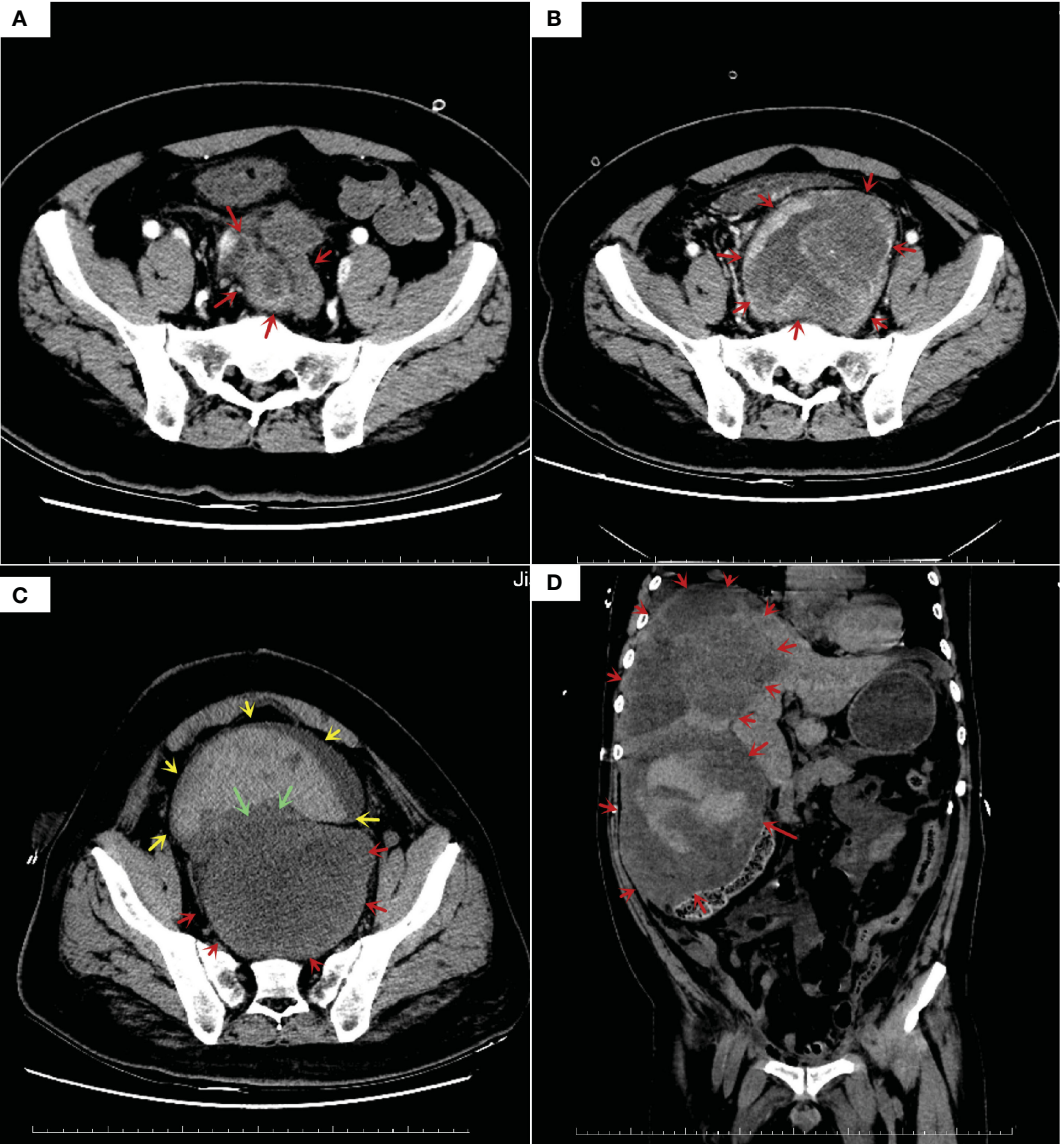
Enhanced computed tomography (CT) results: **(A)** CT before treatment showed a 8.6*11.4*12.4 cm tumor in pelvic, prostate, seminal vesicle gland and bladder was squeezed; **(B)** Follow-up CT examination at 5 weeks after oral imatinib therapy showed that the tumor volume doubled in size (10.0*12.4*20.5cm), rectum squeezed newly; **(C)** Follow-up CT examination at 1 weeks after laparoscopic ileostomy showed that no growth in tumor size, with overfilled bladder and massive blood accumulation; **(D)** Follow-up CT examination at 3 months after total pelvic organ resection showed multiple hypodense nodules and masses in the liver S4/8, larger ones (14.4*12.5*11.6 cm).

In 2022, a 50-year-old patient was admitted to the hospital for surgery for a perianal abscess. During clinical examination, a large mass measuring 8.6cm*11.4cm*12.4 cm (**Figure 1A**) was seen in the pelvic cavity. The puncture pathology findings were suspicious of pelvic mesenchymal tumor. Microscopically, the mass consisted of ovoid or spindle-shaped cells arranged in a bundle or woven pattern, some of which had a translucent cell pulp and large dark-stained nuclei, with scattered nuclear fission images visible, surrounded by the prostate body and smooth muscle tissue. Immunohistochemistry (IHC) of the puncture results revealed Vimentin and β-Catenin positivity, CD34 and DOG1 partially positivity, SMA, CD117, Desmin, S-100, HMB-45 and Bcl-2 negativity, with Ki-67 about 60% positivity. The molecular pathology results showed that c-KIT (exon9.11.13.17) and PDGFRα (exon12.18) were not mutated, with no MDM2 amplification detected by FISH. Based on the conventional pathology, IHC, FISH and generation sequencing of the puncture lesion, the diagnosis of “malignant spindle cell tumor” could only be given, and such diagnosis did not make specific pathological typing, which would make our determination of clinical treatment plan difficult.

In May 2022, a dose of 400 mg imatinib capsule was given orally for antitumor treatment, CT findings 2 months later showed tumor progression and doubling of tumor size (**Figure 1B**), then treatment was changed to 50mg Sunitinib, the tumor was not effectively controlled (**Figure 1C**), half a month later there was overfilling of the bladder, effusion in the thorax and pelvis, and even acute renal failure.

In August 2022, total pelvic resection was performed at another hospital, and one month after surgery, multiple hypodense nodules and mass shadows in the liver, the largest one was 14.4cm*12.5cm*11.6 cm, massive right pleural effusion and compressive pulmonary atelectasis, and metastases in both lungs were not excluded as a possibility (**Figure 1D**).

**Figure 2:**
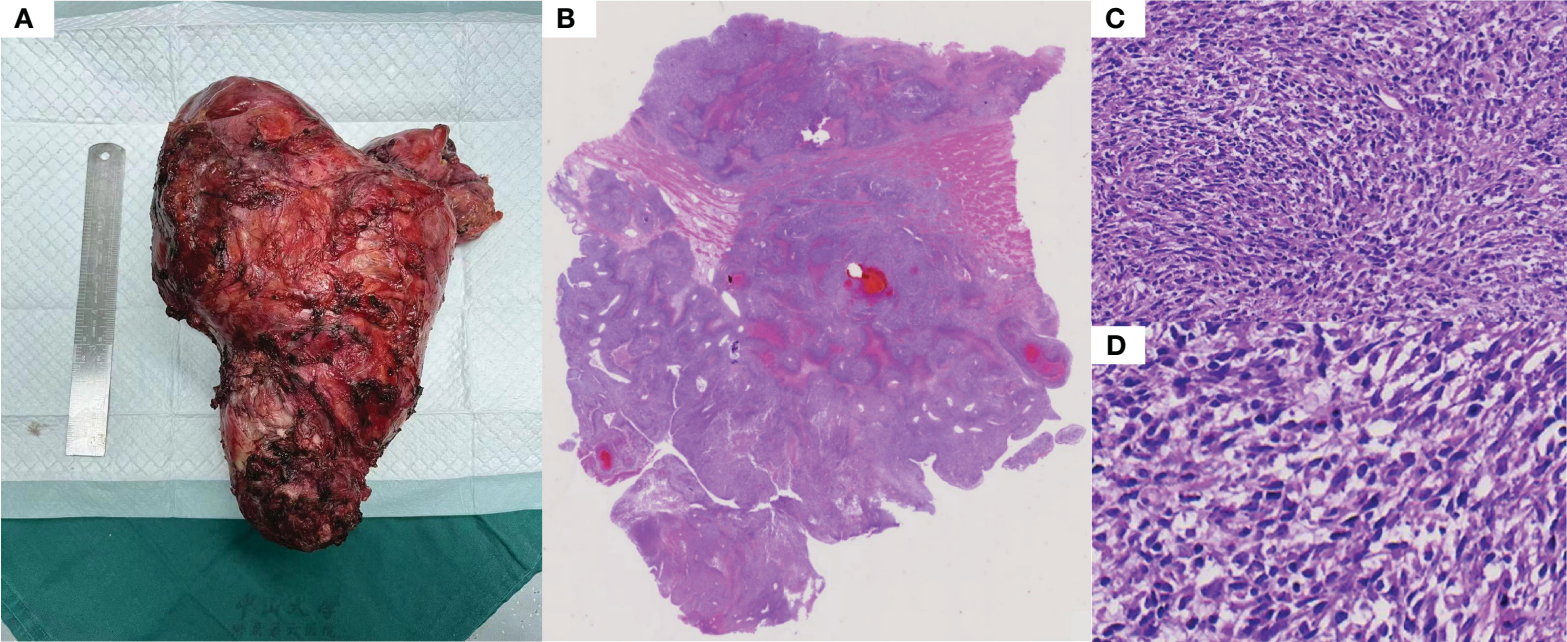
Histopathological features: **(A)** Large mass removed by total pelvic resection. **(B)** Overall the histopathology. **(C)** The tumor was composed of ovoid or spindle-shaped cells, arranged in a bundle or woven pattern (HE-100X). **(D)** There are some distinct areas of interstitial collagen fiber proliferation, some of which are visible as hemorrhagic necrosis (HE-100X).

**Figure 3:**
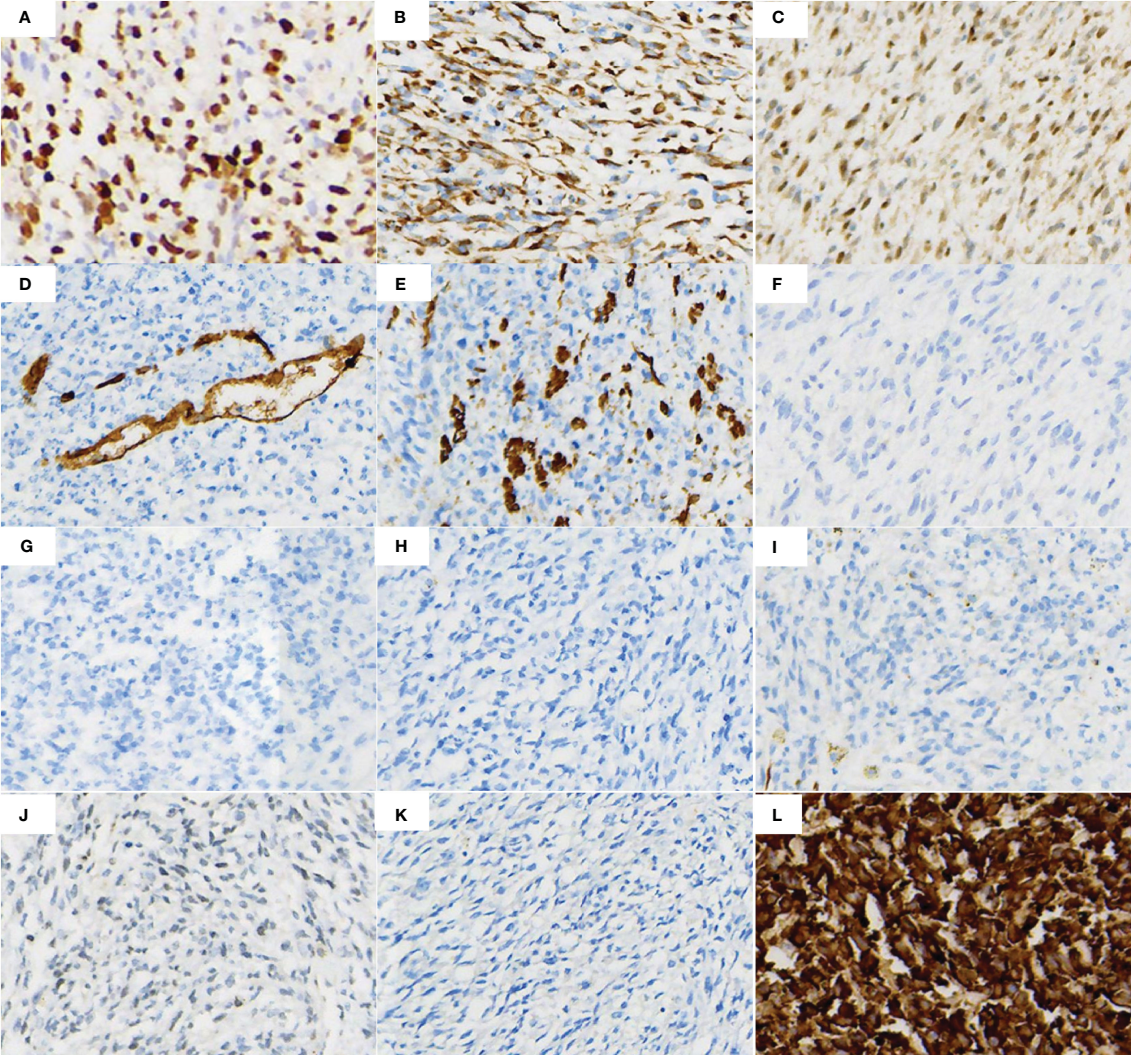
IHC markers staining of the surgical tissue cells showed **(A)** Ki-67 proliferation index in most of the tumor areas was around 60%, **(B, C)** Vimentin and CDK4 positivity, **(D, E)** CD34 and SMA partial positivity, **(F–K)** ALK, EMA, S100, Desmin, CD117 and DOG1 protein negative expression, **(L)** Pan-TRK positive expression.

Total pelvic resection specimen showed a huge mass (29cm*17cm*11cm) in the pelvis, the cut surface showed cystic solid mass, tough texture, focal necrotic pattern (**Figure 2A**). Microscopically, the tumor cells were more uniformly short spindle-shaped and diffusely distributed, with high cell density and infiltrative growth (**Figure 2B**). Some of the tumor had vacuolated cytoplasm, and the tumor cells were obviously heterogeneous, with large deep-stained nuclei and a mitotic rate of >5/10 high-power fields (**Figure 2C**). Additionally, interstitial collagen fibers were obviously proliferated, and hemorrhagic necrosis was seen in some areas (**Figure 2D**). The IHC analysis of surgical specimen showed that the proliferation index (Ki67) is roughly 60% (**Figure 3A**). Besides, Vimentin and CDK4 were positive in the case (**Figures 3B, C**), while the expression of CD34, S100, Dog1, Desmin, CD117, MDM2, SMA, EMA and ALK were all lost in the tumor (**Figures 3D-K**).

To further verify the true picture of the tumor, further molecular characteristic test was required. Considering the tumor was a malignant tumor of soft tissue origin (sarcoma), surgical tumor samples were further detected by Next-generation sequencing (NGS). The detection results included point mutation, small fragment insertion and deletion mutation, gene fusion and copy number variation, microsatellite instability (MSI) analysis, and tumor mutational burden (TMB). In the sequencing of genes, significant fusion mutation *TPM3-NTRK1* was found in the results with 41.59% mutation abundance, and this result was validated by IHC (**Figure 3L**) and Sanger sequencing (**Supplementary Figure 1**). The *TPM3-NTRK1* fusion involving exons 1–6 of *TPM3* and exons 11–17 of *NTRK1* (**Supplementary Figure 1B** show the fusion mode). The results suggested that the patient may benefit from targeted agents such as entratinib, larotinib, lopatinib and sitravatinib. In addition, NGS results showed other potentially clinically significant mutations as follows: *TERT* (c.-124C>T), *TP53* (c.743G>A), *CDKN2A* (copy number deletion:0.2), *CDKN2B* (copy number deletion:0.3), *MTAP* (copy number deletion:0.3) and two unknown clinically significant mutations. Among them, *TP53*, *CDKN2A*, *CDKN2B* are several important oncogenes, which contributed to the poor prognosis of this patient. Details of location and function are shown in **Table 1**. NGS results also showed a very low TMB (1.99 mutations/Mb) and MSI analysis showed MSS.

**Table 1:** The mutation of genes detected in surgical tissue.

Gene	Mutation	Abundance	Detection method
*TPM3-NTRK1*	T4:N11 fusion	41.59%	RNA-based NGS
*TERT*	c.-124C>T	43.75%	DNA-based NGS
*TP53*	c.743G>A	4.70%	DNA-based NGS
*CDKN2A*	copy number deletion	CN:0.2	DNA-based NGS
*CDKN2B*	copy number deletion	CN:0.3	DNA-based NGS
*MTAP*	copy number deletion	CN:0.3	DNA-based NGS
*DAXX*	c.931C>T	46.48%	DNA-based NGS
*PTCH1*	c.258_260del	83.33%	DNA-based NGS

In conclusion, based on the diagnostic value of *TPM3-NTRK1* fusion variant results from NGS, combined with morphology, IHC and relevant clinical information, we made the final diagnosis: *NTRK*-Rearranged Spindle Cell Neoplasm (high-grade). Unfortunately, the patient eventually died of organ failure 1.5 months after surgery.

## Discussion

Soft tissue sarcoma (STS) is a heterogeneous mesenchymal tumor, NTRK-RSCNs, included in the 2020 WHO Classification of Soft Tissue Tumors, was described as a new STS type with multiple morphologic, histologic gradations and extensive clinical aggressiveness, with *NTRK* fusion as an important molecular characteristic (3). However, inflammatory myofibroblastic tumors (IMT) (5), infantile fibrosarcomas (IFS) (6) and gastrointestinal stromal tumors (GIST) (7) reported with NTRK fusions poses a challenge to the differential diagnosis of NTRK-RSCNs, especially in the absence of adequate morphological immunohistochemical characterization. In this case, disease entities like wild-type GIST caused early diagnostic confusion, despite the c-KIT and PDGFRα was wild type detected by sanger sequencing result.

Histologically, NTRK-RSCNs were consisted of spindle-shaped cells and were generally highly infiltrative (8). Immunohistochemically, NTRK-RSCNs tumor cells show co-expression of S100 protein and CD34 frequently, but also show patchy expression of S100 protein and CD34 (3). In this case, the expression of CD34 was intravascular positivity with negative S100, which indicated IHC markers were not enough to make a differential diagnosis. In addition, among NTRK1-RSCNs, NTRK2-RSCNs and NTRK3-RSCNs, NTRK1- RSCNs span a wide clinical spectrum, with highly variable histology and tumor grade (9). Overfield, C.J et al. reported a low-grade *TPM3-NTRK1* fusion RSCNs with prominent vascularity, conspicuous stromal, perivascular hyaline fibrosis and patchy lymphoplasmacytic infiltrates, the expression of CD34 was diffuse positivity and S100 scattered cells expressing (10). Four *TPM3-NTRK1* fusion RSCNs cases in adolescents reported by Jen-Wei Tsai et al. showed tumor borders focally infiltrative, CD34 and S100 co- expression (11).

Accurate diagnosis of rare tumors is not only dependent on the underlying support of morphology and immunohistochemistry, but also needs to be complemented by relevant molecular characteristics and fully integrated clinical information. In NTRK-RSCNs, other detailed molecular characteristics remain unclear, in addition to the molecular characterization of the NTRK fusions. However, this NTRK-RSCNs case showed *CDKN2A/B* deletion, TP53 missense mutation and other mutation gene of *TERT*, *PTCH1*, *DAXX* and *MTAP*. Among them *CDKN2A/B* deletion and *TP53* mutation have been reported in NTRK-RSCNs (9, 12),which implies worse prognosis. *TERT* promoter mutation is a central event in tumor progression and prognostic marker in chondrosarcoma (13), thyroid carcinoma (14) and central nervous system tumors (15). In this case, *TERT* promoter c-124C mutation was first reported in NTRK-RSCNs, which may increase the expression of mRNA. *PTCH1* is a part of the hedgehog signaling pathway involved in tumorigenesis (16). *MTAP* is a polyamine metabolism-related gene whose mutations are associated with poor prognosis in a variety of tumors (17, 18). Methylation of DAXX implicated in sarcoma progression (19), interestingly, the NTRK-RSCNs harbor a unique methylation class that could distinguishes them from entities such as IFS, IMT and malignant peripheral nerve sheath tumors (MPNST) and so on. According to a DNA‐methylation based classifier study about sarcomas, eight of NTRK-RSCNs cases share a common DNA‐methylation profiling known to be correlated with a distinct cell of origin (8). In summary, further series are needed to explore the specific molecular characteristics of NTRK-RSCNs.

Identification of *NTRK* fusion tumors has been proven to be meaningful because chemotherapeutic NTRK inhibitors, including Entrectinib and Larotrectinib, showed the efficacy and safety in treating advanced *TRK* fusion sarcomas (20). Huang W K et al. newly reported a patient with locally advanced and *TPM3- NTRK1* fusion pelvic spindle cell tumor, who responded well to neoadjuvant Larotrectinib therapy, after 4 months of adjuvant Larotrectinib therapy, the patient remained disease-free for 8 months and underwent successful surgical resection (21).

In conclusion, we report a novelty case of high-grade NTRK-RSCNs with *TPM3-NTRK1* fusion occurring in the pelvis. Firstly, Although NTRK-rearranged spindle cell neoplasms have been reports before (21, 22), this case with different clinical and molecular characteristics. This case represents the first reported instance of imaging, pathology, molecular characteristics, and clinical features integration. *TRK*-targeted drugs such as Larotrectinib could be potential targeted therapy. Secondly, soft tissue origin tumors (sarcoma)specially involve multiple fusion genes, which usually has a high degree of morphological overlap, and that makes it difficult for traditional techniques such as IHC, FISH, and RT-PCR to meet the increasingly sophisticated molecular typing needs. The implication of this case is that when soft tissue spindle cell tumors cannot be accurately diagnosed by morphology and immunohistochemistry, especially when the nature of the tumor is difficult to be determined, molecular characterization by NGS is highly recommended, which can help us to clarify the diagnosis and may help to identify and treat the relevant target genes. The “integrated” pathology diagnosis, including pathology morphology, immunohistochemistry, various molecular characteristic and clinical information, will play an increasingly important role in the pathology diagnosis of complex tumors and the selection of therapeutic targets.

## Data availability statement

The original contributions presented in the study are included in the article/**Supplementary Material**. Further inquiries can be directed to the corresponding authors.

## Ethics statement

The studies involving humans were approved by Ethics committee of Wuyi Traditional Chinese Medicine Hospital. The studies were conducted in accordance with the local legislation and institutional requirements. The human samples used in this study were acquired from a by- product of routine care or industry. Written informed consent for participation was not required from the participants or the participants’ legal guardians/next of kin in accordance with the national legislation and institutional requirements. Written informed consent was obtained from the individual(s) for the publication of any potentially identifiable images or data included in this article.

## Author contributions

QC: Data curation, Formal analysis, Writing – original draft, Writing – review & editing. ZH: Data curation, Formal analysis, Writing – review & editing. HL: Data curation, Writing – review & editing. XH: Formal analysis, Writing – review & editing. LW: Methodology, Writing – review & editing. YY: Methodology, Writing – review & editing. BL: Writing – review & editing. JH: Conceptualization, Project administration, Writing – review & editing. JG: Conceptualization, Project administration, Writing – review & editing.
