# Supplementary material for: Case report: Adult NTRK-rearranged spindle cell neoplasms with TPM3-NTRK1 fusion in the pelvic
Source: Front Oncol. 2024 Jan 31;14:1308916. doi: 10.3389/fonc.2024.1308916 (PMC10864579; doi:10.3389/fonc.2024.1308916)
Supplement: Supplementary file 1 [file DataSheet_1.docx]

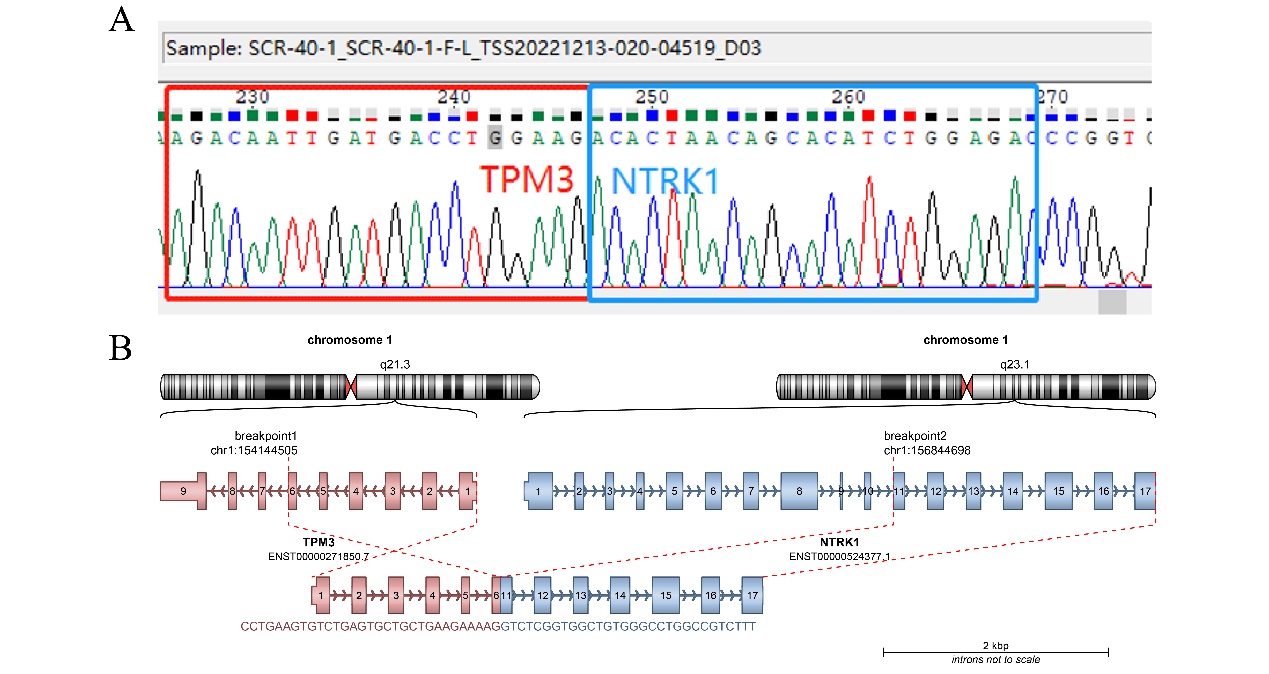


Supplementary Fig.1 Sanger sequencing validation of TPM3-NTRK1 fusion(A) and fusion mode of TPM3-NTRK1 fusion(B)
